# Emotional needs for smart products: a case study of older people living alone in Chengdu, China

**DOI:** 10.3389/fpsyg.2025.1584543

**Published:** 2025-05-26

**Authors:** Liu Yun, Rosalam Che Me, Irwan Syah Md Yusoff

**Affiliations:** ^1^Department of Product Design, Faculty of Fine Arts and Design, College of Chinese & ASEAN Arts, Chengdu University, Chengdu, China; ^2^Department of Industrial Design, Faculty of Design and Architecture, University Putra Malaysia, Serdang, Selangor, Malaysia; ^3^Malaysian Research Institute on Ageing (My Ageing), University Putra Malaysia, Serdang, Malaysia; ^4^Department of Resource Management and Consumer Studies, Faculty of Human Ecology, University Putra Malaysia, Serdang, Selangor, Malaysia

**Keywords:** ageing, older people living alone (OPLA), emotional needs, smart product, thematic analysis

## Abstract

Older people living alone (OPLA) face significant challenges in maintaining emotional well-being, especially in the context of rapid urbanization and social change. Smart products are increasingly viewed as promising tools to support healthy and independent aging. This study explores the emotional needs of urban OPLA in Chengdu, China, and examines their perceptions and use of smart products as emotional support tools. Using a qualitative approach, in-depth interviews were conducted with 20 OPLA aged 60 and above. Thematic analysis revealed three key themes: (A) Emotional well-being of OPLA due to living alone, (B) Efforts to satisfy emotional needs, and (C) The use of smart products to satisfy emotional needs. While many older adults adopt self-regulatory strategies and express interest in technology, low motivation and usability barriers hinder their engagement with smart products. This study highlights the need for emotionally responsive, user-friendly, and culturally attuned smart technologies. The findings offer theoretical and practical insights into promoting smart aging through inclusive design and inform policies aimed at improving emotional well-being among older adults living alone.

## 1 Introduction

According to the World Health Organisation (WHO), one-fifth of the global population will be over the age of 60 by 2050. (United Nations, 2020). As the world’s second-largest economy with a population of 1.4 billion, there will be 400 million people over the age of 65 in China by 2050 (Fang et al., 2015). With the increasing prevalence of the aging population, as well as changes in social and family structures, an obvious trend of family miniaturization has emerged, with a significant increase in the number of older people living alone (OPLA) or empty nesters, amounting to 118 million in China in 2015 (Han et al., 2020). Studies have shown that OPLA patients are more likely to suffer from social isolation, loneliness, depressive symptoms, and suboptimal social support (Kawamoto et al., 2005). While living alone in China may be a response to improved housing quality and abundant alternative living arrangements, it is critical to examine how older Chinese people cope with their emotional needs in the face of such changes. As the physical and perceptual abilities of older people regress, their main needs shift from material needs centered on caregiving to spiritual needs centered on socialization, respect, and self-actualization. Western scholars refer to these needs as the “3Ms,” i.e., money, medical, and mental needs, which represent financial, medical, and spiritual needs (Mo, 2010). Therefore, as part of spiritual needs, emotional well-being is an integral part of older adults’ well-being (Malatesta and Kalnok, 1984).

In literature, there is a rich body of knowledge on the daily life experiences of OPLA from other countries. The British Geriatrics Society (British Geriatrics Society (BGS), 2017) considers OPLA a vulnerable group, stating that OPLA requires health, housing, financial assistance, transportation, and social care to maintain a healthy lifestyle and enjoy it later. Similarly, a Korean study revealed that older adults living with relatives scored significantly higher on several physical and mental health indicators than did OPLA adults (You and Lee, 2006). In contrast, an Australian study reported the benefits of living alone, as the time provides an opportunity for self-reflection and spiritual activity (Stanley et al., 2010).

In qualitative research, reality is constructed within cultural, historical, and social contexts (Korstjens and Moser, 2017). Owing to the differences in cultural context and lower socioeconomic levels between China and the West, research should be conducted to explore the daily lives of OPLA in the Chinese context. Culturally, older Chinese adults are not expected to live alone, as the family remains the traditional provider of old-age care. In contrast to Western communities, solitary living arrangements are associated with more stigma (Jia et al., 2023) and greater levels of loneliness (Gu et al., 2019; Chou and Chi, 2000) in China. Therefore, older people living alone face greater challenges in maintaining their physical and mental health in China. Moreover, with the development of science and technology, smart products have shown some potential in meeting the physical and mental needs of the elderly population.

The advent of smart home technologies in recent years has offered a promising solution to help older adults stay at home and live independently while maintaining good quality of life at an affordable cost and with minimum human resource requirements (Curumsing et al., 2019). Smart products contain sensory and computing capabilities to conduct interactive activities with people at the physical level and emotional level (Luo et al., 2023). However, despite the growing literature on ageing and technology, few studies have combined interdisciplinary theoretical frameworks (e.g., social gerotechnology and affective design theory) to examine how OPLAs in urban China perceive and emotionally interact with smart products in their daily lives. Existing research has tended to focus on physical or safety needs, ignoring the emotional dimensions and cultural contexts that influence the adoption of technology in older populations, and emotional neglect in product design can lead to rejection or underutilization of technology. Therefore, this study provides design insights for emotionally intelligent and culturally resonant smart products by capturing the personal experiences of OPLA in a Chinese city, informing inclusive policies and service systems that truly meet the multifaceted needs of this vulnerable population. The findings can be used to make recommendations on promising practices and policies, such as improving the design of relevant smart products, measures, and services through theories such as user experience design or emotional design to meet the emotional needs of OPLA. Thus, two related research questions were posed: (1) What strategies did urban OPLA take to meet their emotional needs in their daily life experiences? (2) What was the attitude of urban OPLA toward using smart products to meet their emotional needs?

## 2 Materials and methods

### 2.1 Literature synthesis

In recent years, there has been a growing academic interest in the intersection between smart products and emotional wellbeing, particularly in an ageing society. Research has shown that smart technologies, from home assistants to interactive devices, contribute not only to the physical health of older people, but also to their psychological and emotional lives (Ghorayeb et al., 2021). Smart products equipped with sensing and computing capabilities enable two-way interactions that enhance the user’s sense of companionship, control, and autonomy (Lucia-Palacios and Pérez-López, 2021). However, the affective efficacy of these technologies remains underexplored, especially for older adults living alone in non-Western cultural contexts.

OPLA face significant psychological needs related to social connectedness and emotional well-being (Hou and Zhang, 2023; Abell and Steptoe, 2021), which are exacerbated by technological and access barriers (Peek et al., 2014). Hong et al. (2022) highlights a global research trend, noting that assistive technology-equipped smart homes can help meet a variety of needs of older people, including emotional, social and physical fulfilment. Research suggests that emotional and physical usability are critical; older adults need environments that not only facilitate daily tasks but also evoke a sense of security and belonging (Lee and Kim, 2020). In addition, trust and acceptance are key barriers to the adoption of smart technologies by older adults. Lou and Hong (2023) found that perceptions of trust, ease of use, and affordability strongly influence technology acceptance. Expressed the same view, noting that older adults in China are ready to adopt smart care products but still face challenges related to emotional connection and perceived utility. For example, in less developed regions such as the west, efforts are needed to increase digital literacy and user engagement with smart care technologies, reflecting broader social support systems (Wang et al., 2024). Whilst digital technologies provide valuable tools to enhance social interactions, issues such as low digital literacy, lack of confidence in operating smart devices, and concerns about privacy or trust are still prominent. Overcoming these barriers requires multifaceted strategies that emphasize improving digital skills, improving access, and fostering positive attitudes toward technology adoption among this vulnerable group (Tsai et al., 2015; Raihan et al., 2024; Pérez-Escolar and Canet, 2023).

To better understand how design can address these issues, emotional design and emotional health frameworks are increasingly being adopted. Emotional design focuses on the creation of products and interfaces that promote positive emotional responses, while emotional health addresses an individual’s overall emotional well-being and life satisfaction. Integrating these concepts can significantly improve the quality of life of older people through supportive technologies and interventions. Norman (2004) emphasizes that good design should appeal to the emotional and cognitive dimensions of the user, influencing usability and satisfaction. Peine et al. (2021) propose Socio-Gerontechnology to understand the interaction between older people and technology from a sociological and philosophical perspective of science and technology, emphasizing that technology and ageing are co-constructed. In addition, Morville (2005) UX Honeycomb framework provides a structured approach to assessing how users interact with a product along seven dimensions: useful, usable, desirable, findable, accessible, trustworthy and valuable. These dimensions provide a holistic perspective for evaluating older users’ experience of smart products, particularly in relation to their emotional resonance and perceived value. The implications of emotional design extend to interventions aimed at improving emotional well-being. For example, horticultural therapy and intergenerational approaches have shown promise for improving the emotional health of older adults (Han et al., 2018; Whear et al., 2023). By applying affective design principles to such interventions, it may be possible to foster closer emotional connections and avoid feelings of loneliness and depression, thereby promoting overall well-being. In summary, the intersection of emotional design and emotional well-being provides an opportunity to effectively support older adults living alone. By incorporating emotional design principles into technological solutions and emotional interventions, it may be possible to improve quality of life, encourage social interactions, and promote emotional resilience in this vulnerable population.

Despite the growing body of related literature, few studies have combined these theoretical frameworks with qualitative, in-depth research on how urban Chinese OPLAs perceive and emotionally interact with smart products in their daily lives. At the same time, the intersection of smart technologies with the emotional needs of older adults requires a nuanced approach that encompasses usability, emotional support, and community engagement. This study aspires to continue to bridge the gap in understanding these dynamics to better serve the aging population at home and abroad.

### 2.2 Study design

This study used qualitative research methods to conduct in-depth face-to-face interviews. This study was conducted in accordance with relevant ethical guidelines and regulations, including the Declaration of Helsinki and institutional ethical standards. Ethical approval for this study was obtained from the Ethics Committee for Research Involving Human Subjects University Putra Malaysia (Approval No.: JKEUPM-2024-673).

All participants provided informed consent before participation. They were informed of the research objectives, procedures, and their right to withdraw at any time without consequences. To ensure confidentiality, all identifying information was removed from the data, and anonymous codes were used during analysis.

#### 2.2.1 Settings and participants

**Figure 1:**
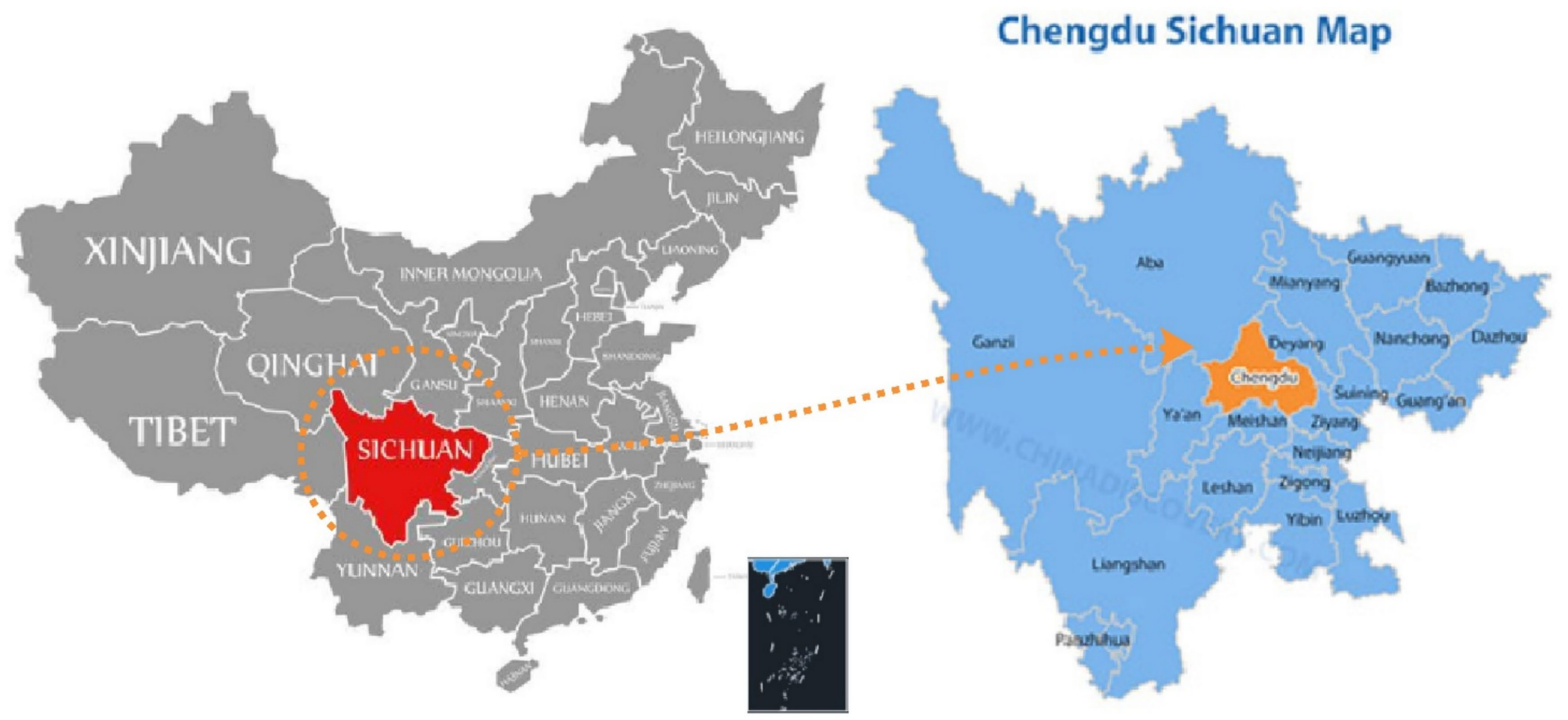
Study site (redrawn and annotated by the authors).

The study was conducted from June 2024 to July 2024 in Chengdu, an inland city in China. It is the core city of the Chengdu–Chongqing region’s twin-city economic circle, with an urbanization rate of 74.4% (Chengdu Municipal Bureau of Statistics, 2020). The city of Chengdu was selected for three main reasons: (1) Chengdu is located in the heart of Southwest China (Figure 1). It is the capital of Sichuan Province and one of the largest cities in China (Wang, 2012), with a deep history and culture. (2) A high degree of globalization whereby 252 of the world’s top 500 companies had established operations in Chengdu by the end of 2013. The rapid globalization process introduced Western culture and ideas to local people. (3) Aging phenomenon: According to the 6th census, the elderly population (over 65 years old) accounts for 9.7% of the total population (Banister et al., 2012), and the old-age dependency ratio (ODR) is reported to be 16.3. Thus, the elderly population in Chengdu is 2.1% greater than the national population (Banister et al., 2012). A high proportion of the OPLA stay in the city, some in a restricted living environment, hence likely giving rise to some problems.

Five main urban areas in Chengdu (Jinniu, Qingyang, Wuhou, Chenghua, and Jinjiang) with prominent aging populations were selected as the study area. These five areas were home to 992,815 elderly people aged 60 years and above, accounting for 26.4% of the overall population in Chengdu (Zhang et al., 2023). Participants were selected through purposive sampling (Lincoln and Guba, 1985) to recruit older adults living alone in urban communities. To ensure diversity in experiences, a maximum variation sampling strategy (Patton, 1990) guided the inclusion of individuals across age, gender, socioeconomic status, and duration of solitary living (1–25 years). Collaboration with local stakeholders including social workstations, neighborhood committees, community health centers, and grassroots organizations facilitated participant identification. Screening was carried out by trained community workers recruited through short interviews during the organization of community events.

They were recruited while they were participating in community activities organized by the above organizations or as members of the case consultation.

**Figure 2:**
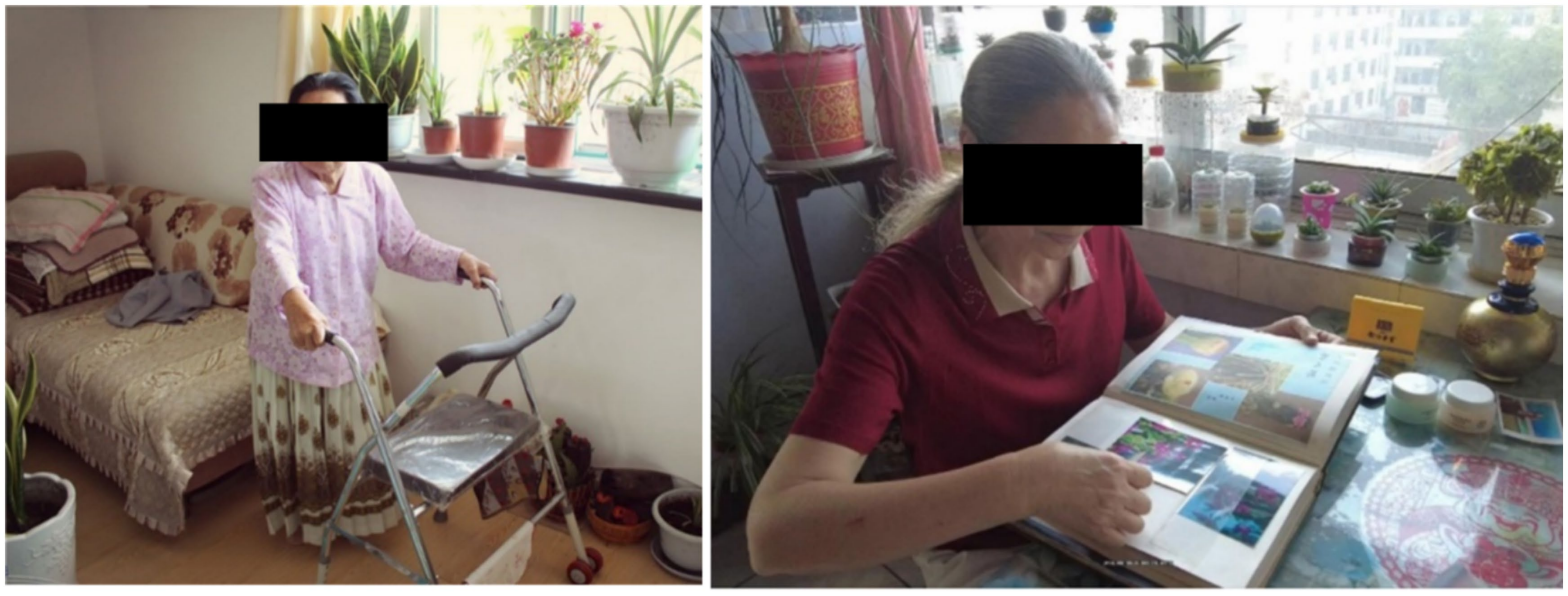
Interview photos (partial).

The interviews were conducted in separate meeting rooms or in the homes of OPLA to ensure a quiet and private environment (Figure 2). The inclusion criteria included older adults who (1) were 60 years of age or older; (2) lived alone or, if they had children, were not in the same community as their children and received a low frequency of caregiving; (3) had basic mobility; and (4) had comprehension and were able to communicate and express themselves comfortably. The exclusion criteria for the subjects were as follows: (1) living alone but in the same community as their children, with a high frequency of care, and (2) having cognitive disabilities or being unable to communicate normally.

#### 2.2.2 Instruments

The interview guide was developed based on a literature review (Minichiello et al., 2008) and refined by researchers and community workers. It contains three main sections: (1) Demographic and background information about the older person; (2) OPLA’s daily life experiences and emotional needs; and (3) Perceptions and attitudes of OPLA toward using smart products to fulfill emotional needs. Please see Table 1 for a detailed interview outline. Each interview was recorded and transcribed verbatim. The interviews lasted between 45 and 60 min.

**Table 1:** Interview outline.

Question category	Question content
Section1. Demographic and background information	Name, gender, age group, marital status, education level, occupation, living arrangements, Residential Stability, Social Interaction with Family, Caregiving and Support System.
Section 2. Daily life experiences and emotional needs	Can you describe a typical day in your life? What kind of activities do you usually participate in during the day or week? Do you attend any community activities or social events? If so, how often and what kind? Do you often feel lonely or emotionally distressed? When do these feelings occur most frequently? What strategies or habits help you feel emotionally better or more connected? Do you have any hobbies or personal routines that bring you joy or comfort? What kinds of emotional support (if any) do you wish you had more of?
Section 3. Attitudes toward using smart products for emotional support	Are you familiar with or have you used any smart products at home? Can you describe your experience using these products? What do you like or dislike about them? Do you use any technology to communicate with your family or friends? Do you think smart products can help meet your emotional needs? Why or why not? What challenges do you face when using smart products? What kind of smart product features would make you feel more emotionally supported or accompanied? If you could design your own smart product to support your emotional well-being, what would it look like or do?
Section 4. Closing questions	Is there anything else you would like to share about your daily life or how technology could help improve your emotional well-being? Would you be willing to participate in future follow-up interviews or product design workshops?

#### 2.2.3 Data collection and analysis

In-depth interviews, a commonly used method in qualitative research, rely on the informative power of the interview process (Malterud et al., 2015). The sample population should be neither too small nor too large (Kvale, 1996; Sandelowski, 1995). Saturation is often mentioned as a criterion for sample size determination in qualitative research (Saunders et al., 2018). Saturation occurs when the researcher no longer receives information from the participants that adds to the theory that has been developed (Malterud et al., 2015). No new themes emerged after the 18th interviewee in the coding process, and two more were subsequently interviewed. The sample size for the qualitative interviews (*N* = 20) was determined based on the point at which thematic saturation was achieved (Guest et al., 2006). Specifically, thematic saturation was monitored through an iterative coding cycle in which two researchers independently coded transcripts after every five interviews and conducted intercoder comparisons. Saturation was considered to have been reached when new interviews yielded repeated codes and redundant themes across key emotional and behavioral dimensions (Saunders et al., 2018).

Prior to the interview, the researcher purposefully screened the OPLA sample by contacting community staff. The research team comprised three members: an inclusive design researcher (PhD candidate, female, 10 years of experience in inclusive design studies), a social work practitioner (MSW, male, 5 years of experience), and a student on industrial design (Master candidate, female). To minimize bias, all researchers documented their preconceptions about the topic in reflexive journals prior to data collection. Regular team discussions were held to critically examine how personal experiences might influence data interpretation. No conflicts of interest were identified. During the session, one researcher led the questioning, while the other two focused on notetaking and recording.

**Figure 3:**
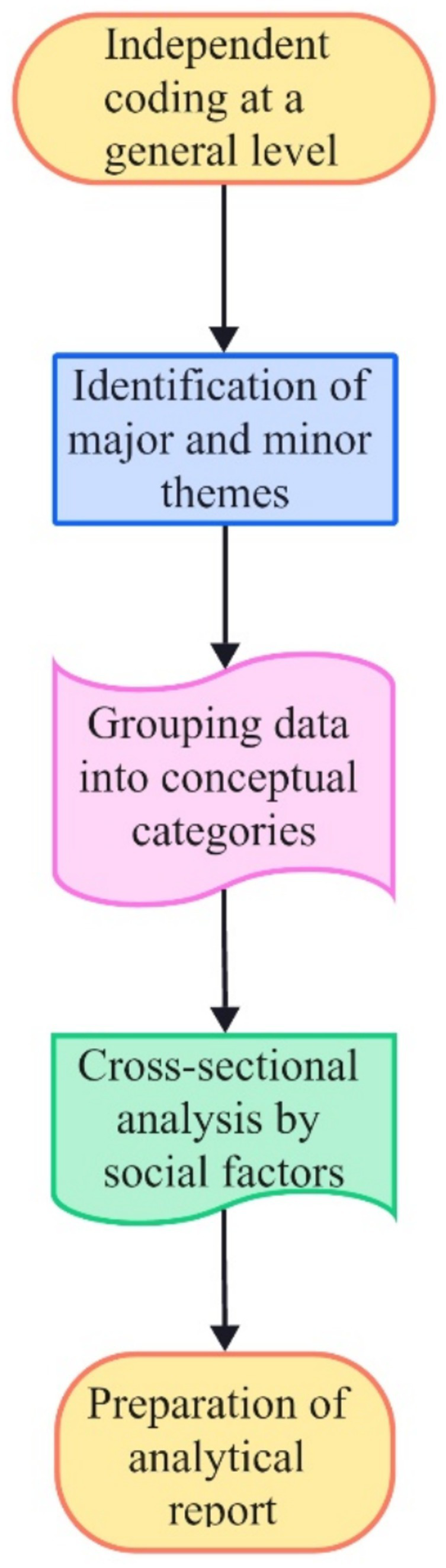
Steps in thematic analytic process (adapted from Braun and Clarke, 2006, 2012).

All participants provided written informed consent to participate in the study. Following informed consent, semi structured in-depth interviews were conducted. Examples of vivid and convincing excerpts were selected and analyzed. The transcripts were uploaded to ATLAS.ti (ATLAS.ti version 23, 2018) and analyzed because of “A Method for Identifying, Analyzing, and Reporting Patterns (Themes) in Data” (Braun and Clarke, 2006). There were several stages of data analysis (Figure 3): (a) Independent coding at a general level, such as applying user profiling to compress the data into analyzable units; (b) identification of major and minor themes discussed during the interviews; (c) analysis that involved grouping the thematic data through the creation of conceptual categories; (d) cross-sectional analysis that tabulates the collected data with various social factors (e.g., gender, age, socioeconomic conditions, and educational background) into a table; and finally, (e) preparation of the results of the analysis on the basis of the research questions and literature review into an analytical report.

Each transcript was analyzed simultaneously via a descriptive qualitative approach to add depth and quality to the data analysis (Vaismoradi et al., 2013). To ensure the validity of the findings, a method of data triangulation, which Denzin (1978) labeled researcher triangulation, was used throughout the analysis, whereby each interview was analyzed by two lead researchers (Charpentier and Kirouac, 2022). Finally, the findings were discussed among all the researchers and then validated against the results of researcher triangulation.

## 3 Results

### 3.1 Sample characteristics

The study outlines the heterogeneous sociodemographic characteristics in terms of the family background, cultural background, and socioeconomic conditions of the study participants, shown in Table 2 sample characteristics (*N* = 20). Their ages ranged from 60 to 85 years (mean 73.5 years), with 55% (=11) of them aged 60–79 years and 45% (=9) aged 80 years and over. More than half of them were female (55.0%), and 45% were college educated. Most participants (*n* = 18; 90%) were widowed, whereas 5% (= 1) were divorced or separated.

**Table 2:** Sample characteristics (*N* = 20).

Demographics	Number or % of the participants
Age	
60–69	2
70–76	8
77–84	9
85 and above	1
Gender	
Women	11
Men	9
Marital status	
Married	18
Other marital status	2
Education	
Primary school	3
junior or high school	12
College or higher	5
Monthly income (CNY)	
RMB 2999 or lower	7
RMB 3000–4,999	6
RMB 5001 or higher	7
Number of children	
0	3
1	10
2	5
3	2
Housing conditions	19
Multistory apartments	14
High-rise apartments	6

100 RMB = 14.53 USD.

In terms of health status, 20 of them reported chronic physical health problems (e.g., coronary heart disease, diabetes, osteoarthritis, and myocardial ischemia). One patient had limited mobility due to hemiplegia. Most of the respondents (70%) lived in multistory apartments, whereas the remaining 30% lived in high-rise apartments. Some stayed on the first floor with a garden. A few of them who lived on higher floors seldom went downstairs due to the lack of elevators or mobility problems; thus, they had to rely on their children to buy supplies for them.

### 3.2 Qualitative findings

**Figure 4:**
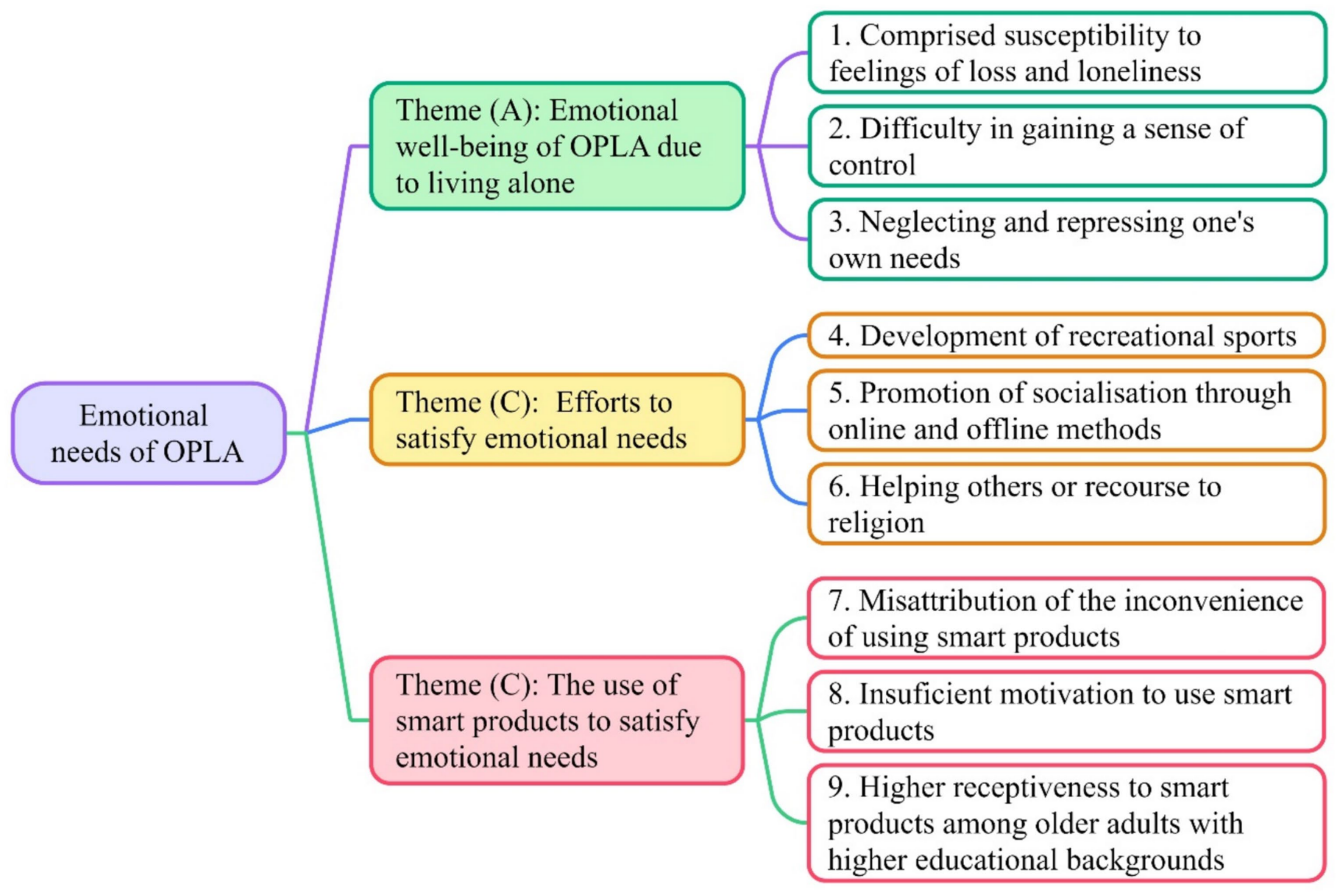
Diagram illustrating interconnected themes and subthemes related to the emotional needs of the OPLA.

The three main themes from the qualitative interviews were (A) the emotional well-being of OPLA due to living alone, (B) efforts to satisfy emotional needs, and (C) the use of smart products to satisfy emotional needs. The themes and subthemes related to the emotional needs of OPLA are summarized in Figure 4, and discussed in more detail below, together with illustrative quotes from the participants.

#### 3.2.1 Theme (A): emotional well-being of OPLA due to living alone

The first theme captures the emotional vulnerability experienced by OPLA, which is shaped by their unique living arrangements. The definition of the community is more specific among OPLA. However, in some instances, the community cannot take care of all the OPLA due to the special nature of the community. For example, some of the study participants stayed in different communities from their children, who seldom visited them. Some OPLAs were unmarried and required different types of attention and care. Overall, most of them yearned to receive some attention from their children or relatives and friends so that they could feel needed and have a sense of belonging.

##### 3.2.1.1 Subtheme 1. Susceptibility to loss and loneliness

Many participants reported chronic feelings of emotional emptiness, isolation, or abandonment, often triggered by widowhood, child estrangement, or shrinking social circles. Loneliness was a dominant emotion that negatively affected their mental health (Victor and Yang, 2012).

“Our biggest need is that we want someone to come to see us and talk to us. Every day, apart from watching TV, we do not have the chance to talk to others. Less communication makes us prone to brain atrophy.” [Grandpa Fei, 75], lives alone in his home, and his son comes back once a year as he works in another province.

“It is just that sometimes I stay at home and feel bored. My children sometimes call me to ask me to wrap some dumplings, so I wrap them and freeze them in the freezer and wait for them to come.” [Grandma Song, 74, whose two children meet her at home weekly].

Home is the place where OPLAs have lived for most of their lives. When their children grow up and leave or their partners pass away, their homes can change from a lively and joyful appearance to an “island.” When OPLAs see things that remind them of their loved ones, they can evoke a strong sense of loss and loneliness.

##### 3.2.1.2 Subtheme 2. Difficulty in gaining a sense of control

With worsening health, it is difficult for elderly people to gain a sense of control, as they live alone at home with no one around them to rely on. Many are very worried about having an accident alone at home without anyone noticing. Most participants felt that they had lived long enough to not fear death but rather how they would die.

“I wish someone would call me every morning and ask if I am still alive.” [Grandma Deng, 80, suffers from coronary heart disease and is seldom visited by her two children who are busy with work].

“I suffer from myocardial ischaemia and worry that I may faint at any time; thus, I hardly ever go out or take public transportation.” [Grandma Wei, 70, suffering from chronic diseases such as myocardial ischemia].

Living alone led some participants to feel a loss of autonomy or helplessness in their daily lives, especially when faced with unexpected events (e.g., illness, accidents), and the lack of immediate support led to emotional insecurity and a reduced sense of autonomy.

##### 3.2.1.3 Subtheme 3. Neglecting and repressing one’s own needs

Many elderly people do not pay attention to their emotional needs, as they accept it as the inevitable process of aging. They also suppress their needs because they do not want to trouble their children and relatives. In traditional Chinese culture, elderly people are more likely to hope for the best for their future generations. When asked about their aspirations, most of the study participants wished for their children and grandchildren to have better future development, often ignoring their needs. As a result of the long-term repression of demand, their mental health and physical health can be neglected.

“Children are busy, and I understand that. I just hope that they live well. My biggest wish is not to disturb them. I can do everything myself.” [Grandpa Wei, 76, whose children are in other provinces and visit only once or twice a year].

Participants often downplayed or ignored their emotional needs, prioritizing pragmatism and survival over self-care. This tendency may reflect generational attitudes and longstanding habits of emotional inhibition that impede emotional expression and coping.

These findings highlight the psychosocial consequences of living alone in old age and emphasize the need for emotional support from OPLA in everyday life.

#### 3.2.2 Theme (B): efforts to satisfy emotional needs

Despite the emotional difficulties described above, many OPLA have developed adaptive strategies to address their emotional well-being. These include physical, social, and spiritual activities.

##### 3.2.2.1 Subtheme 4. Development of recreational sports

In the interviews, the hobbies of the elderly participants included going out for a walk, drinking tea, chatting, playing cards, and mahjong. Elderly people with a higher level of education like to read and listen to audiobooks. Those with reduced mobility tend to watch TV and read news or novels on their cell phones. Some elderly people also spend more time in religious activities, such as worshipping Buddha and chanting sutras. Even though some such as flowers and plants were planted, those remaining in apartment buildings were limited by space availability. However, they resort to growing ornamental or edible plants via ceramic pots or foam boxes on the balcony or security windows.

“I will take care of some flowers and plants, such as ornamental flowers, plums, orchids, etc. I will use my cell phone to look up some knowledge about planting flowers and plants.” [80-year-old Wang Granny is one of the few senior citizens who graduated from a university].

Participation in physical and group activities such as square dancing, walking, and tai chi served not only as a means of maintaining physical health but also as a medium for social interaction and emotional expression. As demonstrated in prior work, such communal activities can enhance mood and mitigate feelings of loneliness among older populations (Cattan et al., 2005).

##### 3.2.2.2 Subtheme 5. Promotion of socialization through online and offline methods

Some communities provide conducive public spaces and infrastructures, as well as organize regular enrichment activities to bring older people together in a group setting. Some of the activities include afternoon social events, weekly happy hours, sewing, knitting, card games, art classes, and games such as mahjong and darts to fulfil the mental needs of the OPLA through social activities. Some seniors also chat with friends through social media, such as WeChat and Jitterbugs, to relieve their boredom. However, this also poses some risks, as seniors are prone to scams.

“The most common topic for old neighbors to get together is to talk about the past, and it is a pleasure to pass the time by chatting at the damper tea stalls.” [Grandpa Fei, 75, lives alone at home with his son returning once a year from another province].

“I will communicate with netizens through WeChat every day to discuss some topics of interest with everyone.” [A 78-year-old retired middle school teacher who lost his son in middle age].

While traditional in-person interactions (e.g., with neighbors or at community centers) remained central to the social lives of participants, some older adults—particularly those with higher digital literacy—also engaged in digital communication via smartphones and online platforms. However, the digital divide remains significant, especially among less educated or older cohorts (Friemel, 2016).

##### 3.2.2.3 Subtheme 6. Helping others or recourse to religion

Elderly people often derive great satisfaction and happiness from helping others. When they recount their experiences of helping others, they become very talkative and happy. Some elderly people also turn to religion in the hope of eliminating their sins and praying for happiness in the afterlife.

“I sponsored some poor college students to complete their studies, and they expressed their gratitude to me through WeChat or cell phone text messages. However, I am contented even if they do not repay me in any way.” [78-year-old retired middle school teachers].

“Sometimes they will pray to Buddha and chant sutras to learn some principles of being a human and to gain peace of mind.” [80-year-old retired employee].

Some respondents found emotional fulfillment through altruistic behavior (e.g., volunteering) or through religious and spiritual practices. These pathways offered participants a sense of purpose and belonging, consistent with socioemotional selectivity theory, which suggests that older adults prioritize emotionally meaningful activities (Carstensen et al., 1999).

These findings suggest that older adults actively seek meaningful engagement and social connections to mitigate the emotional impact of living alone, although their access to coping strategies and preferences vary.

#### 3.2.3 Theme (C): The use of smart products to satisfy emotional needs

The third theme explores the role of smart products in addressing the emotional needs of older adults, revealing both opportunities and barriers. Most of the OPLAs interviewed use a smaller variety of smart products, only use smartphones or smart TVs, or alarms to meet the basic needs of life, as they felt that physical operating interfaces would be easier to operate and that they would be less burdened with information.

##### 3.2.3.1 Subtheme 7. Misattribution of the inconvenience of using smart products

Research has shown that older people often misunderstand or misinterpret technological features due to cognitive decline, leading to difficulties in interaction and subsequent inconvenience or even distress when using smart products (Dermody et al., 2021; Vaportzis et al., 2017). Previous bad user experiences can create a misconception whereby all smart products are bad and expensive among elderly people. They suffer from frustration of not being able to use smart products, believing that it is because of their deteriorating functions. However, in most cases, the root cause lies in the inadequate incorporation of aging-friendly design principles during the product development process.

“Older people are not accustomed to the use of smart products, for fear of operating errors and damage to the items. They are accustomed to old objects in their own homes, so learning to use new products is slightly difficult. The instruction wordings are not readable, and the products are not convenient to use.” [A 77-year-old woman, Granny Deng, widowed 2 years ago, lives alone and has sleep problems].

Many participants struggled with the complexity of digital interfaces and blamed themselves for their inability to operate smart devices. This phenomenon—rooted in low self-efficacy—may result in avoidance behavior, consistent with Bandura’s (1997) theory of self-efficacy, which asserts that perceived personal competence influences behavior and motivation.

##### 3.2.3.2 Subtheme 8. Insufficient motivation to use smart products

The consumption concept among the elderly is still relatively conventional. Even though they may have the ability to consume, they still lack the habit of consuming due to the lifelong habit of thrift and frugality, which manifests as low desire and nonconsumption in the later years of their lives, especially among those who have experienced scarcity in their youth.

“Smart products are expensive and difficult to learn. I am used to my current lifestyle, so it is okay for me not to use it.” [Grandpa Fei, 75 years old, lives alone and his son returns once a year from another province].

In many cases, participants expressed little motivation to engage with smart technologies, citing perceived irrelevance, low trust, or a steep learning curve. Prior studies have similarly highlighted low perceived usefulness and high effort expectancy as critical barriers to technology adoption among the elderly (Venkatesh et al., 2003).

##### 3.2.3.3 Subtheme 9. Greater receptiveness to smart products among older adults with higher educational backgrounds

More than 45% of the study participants graduated from universities. They showed greater acceptance of smart products, and most of them reported a positive user experience for smart products. This could be due to better learning ability to adopt new technology, as well as a higher disposable income that is linked to a greater intention to purchase smart products.

“It would certainly be better if there were similar products. It is just that we know fewer channels and do not know that there are such products that can make life easier. Money is not a problem for me.” [Grandma Liu, 82 years old, was widowed 2 years ago, living alone, with a university degree and a high economic level].

A clear pattern emerged wherein participants with higher levels of education demonstrated more interest and confidence in using smart products. This aligns with findings that education enhances digital literacy and openness to technology-mediated solutions (Charness and Boot, 2009).

These results suggest that while smart technologies hold potential for enhancing emotional well-being in older adults, disparities in education, digital literacy, and motivation significantly mediate their effectiveness.

## 4 Discussion

This study explored how OPLA in Chengdu, China, cope with emotional challenges and how they perceive the potential of smart products in meeting their emotional needs. The findings not only highlight the complex emotional landscape of OPLA but also reveal the opportunities and limitations of current smart products in enhancing their well-being. In this section, the findings are further interpreted through relevant theoretical lenses and discussed in terms of theoretical contribution, practical implications, and future research directions.

### 4.1 Lived experiences of living alone, emotion, and technology among OPLA

#### 4.1.1 Theme (A): emotional well-being of OPLA due to living alone

Theme A highlights the critical role of emotional support from family and friends in the well-being of Chinese older people living alone (OPLA). Rooted in Confucian values, the family remains a primary source of emotional security and social identity in Chinese culture (Luo and Yeh, 2012). Maintaining close connections with children and peers fosters a sense of social belonging, a fundamental human need (Smith, 2017). In contrast, the absence of meaningful social ties often leads to loneliness, depression, and anxiety (Heinrich and Gullone, 2006).

Recent studies confirm that family support correlates positively with mental health and overall well-being in Chinese older adults (Chen et al., 2022), while living with children is associated with higher life satisfaction compared to independent living (Wang et al., 2014). Although living alone may not be emotionally detrimental in some Western contexts (Stanley et al., 2010; Mellor et al., 2008), research shows that in China and other Asian societies, it significantly undermines emotional well-being due to strong cultural expectations around intergenerational cohabitation (Lim and Kua, 2011; Kawamoto et al., 2005; You and Lee, 2006).

Additionally, older adults tend to suppress emotional expression, which may negatively impact on their physical and mental health over time (Carstensen et al., 2000). These findings suggest that emotional support and meaningful relationships remain central to healthy aging, particularly in cultures where family ties are deeply embedded.

#### 4.1.2 Theme (B): efforts to satisfy emotional needs

Under Theme B, OPLA were found to maintain their emotional well-being and sense of independence through leisure activities—voluntary, enjoyable non-work pursuits such as hobbies, exercise, and social interactions (Hills and Argyle, 1998). Participation in such activities has been shown to reduce depression (Fancourt and Tymoszuk, 2019) and enhance happiness and vitality in older adults (Sarid et al., 2010). Engaging with peers and social networks also mitigates loneliness and fosters emotional health (Lee and Ishii-Kuntz, 1987). In various contexts, including the UK and Malaysia, spirituality has been identified as a key dimension of active and healthy aging (Malone and Dadswell, 2018; Tohit et al., 2012).

To sustain independence despite functional decline, tailored support such as assistive technologies and smart products plays a vital role (Buffel et al., 2019). While living alone often leads to social isolation, technologies that facilitate social interaction or help form new connections—such as online support groups—can partially address emotional needs (Tsai and Tsai, 2011; Weinert et al., 2008; Paredes et al., 2021). Although the evidence remains limited regarding the effectiveness of digital interventions for older adults in urban settings (Cohen-Mansfield and Perach, 2015), their potential to support emotional well-being should not be overlooked.

However, age-related emotional characteristics influence user experience. Stereotypes that portray older adults as technology-incompetent may diminish their willingness to adopt new tools. According to Socioemotional Selectivity Theory, as people age, they prioritize emotionally meaningful goals—such as staying connected with family—over knowledge acquisition (Carstensen, 2021). This suggests that emotionally resonant design is more relevant than cognitively demanding interfaces. Moreover, older adults may resist technologies explicitly labeled for seniors, fearing a loss of perceived competence and independence (Astell et al., 2020). In the Chinese context, this aligns with Confucian mianzi (“face”) culture, where preserving dignity discourages use of assistive technologies that imply vulnerability. We describe this tension as “affective-technological dissonance,” underscoring the need to move from “age-friendly” to “culturally and emotionally congruent” design paradigms.

#### 4.1.3 Theme (C): the use of smart products to satisfy emotional needs

Older adults’ misattribution of inconvenience to smart products often stems from a combination of cognitive decline, usability issues, and emotional factors. As cognitive processing slows with age, complex interfaces can become overwhelming, leading to misunderstanding, frustration, and even avoidance (Dermody et al., 2021). These challenges highlight the importance of age-appropriate design, as well as the inclusion of user feedback during development, which has been shown to enhance usability and satisfaction (Borelli et al., 2019).

Moreover, technological anxiety—often rooted in past negative experiences or unfamiliarity—can intensify emotional resistance to smart products (Lou and Liu, 2023). Many older adults desire technology that genuinely improves quality of life but feel alienated by tools that are not designed with their emotional and cognitive needs in mind (Huang et al., 2024). When design fails to support their self-image as independent and competent, particularly in cultural contexts like China’s mianzi (face) culture, it can lead to what we term “affective-technological dissonance.” This highlights the need for a shift from merely “age-friendly” interfaces to culturally and emotionally congruent designs.

Financial concerns also hinder adoption. Many OPLA perceive smart products as expensive or inaccessible, especially in lower-income urban settings (Li and Woolrych, 2021). Although they recognize the potential benefits of technology for aging, they believe only wealthier groups can afford these tools. Government subsidies and community-based support programs are therefore essential to improve both access and motivation (Chen et al., 2022; Kong et al., 2023). Interview data also revealed that older adults with higher education levels exhibited greater emotional adaptability and technological openness, a finding consistent with research on wisdom traits such as empathy, self-reflection, and emotion regulation (Jeste and Lee, 2019). With adequate support—social, emotional, and financial—older adults are more likely to overcome resistance and engage meaningfully with digital solutions that enhance their well-being (Paredes et al., 2021).

In summary, Theme A indicates that living alone generally has a negative effect on the emotional well-being of older adults. This effect is exacerbated by the stoicism and emotional inexpressiveness often observed in older Chinese adults, which further harms their physical and mental health. Theme B highlights that Chinese seniors living alone take proactive steps to maintain their independence despite facing physical and mental risks. Theme C reveals that older adults often blame themselves for difficulties in using smart products, leading to negative experiences such as frustration. However, those with higher educational backgrounds tend to learn more easily and show greater acceptance of these technologies. Therefore, incorporating elements that address the emotional needs of seniors into smart products is crucial. This approach enhances the perceived usability and overall user experience, conveys positive product semantics, and enables older adults to seamlessly benefit from technological advancements.

### 4.2 Practical revelations

**Figure 5:**
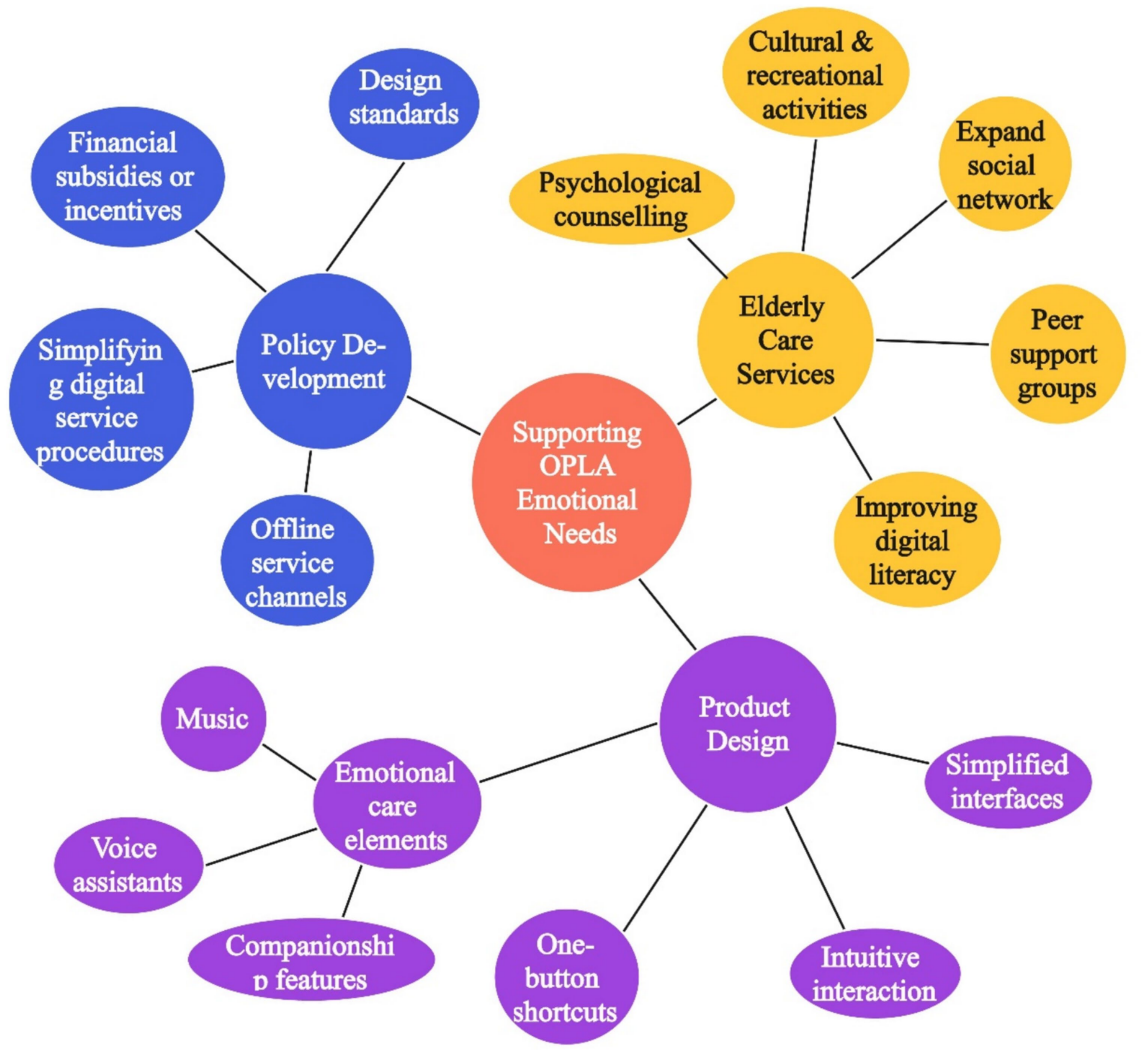
Multi-level strategies for supporting the emotional needs of OPLA.

The findings of this study offer valuable guidance for improving smart product design, enhancing elderly care services, and informing policy development, as shown in Figure 5. The emotional needs of older people living alone deserve urgent attention, as social isolation and loneliness can significantly affect their physical and mental health, posing a growing public health concern.

To begin with, in the realm of smart product design, a human-centered approach should be prioritized to reduce the barriers older adults face when using digital technologies. Simplified interfaces, intuitive interaction processes, and features such as one-button shortcuts or “elderly-friendly” modes can minimize frustration and improve accessibility (Wang et al., 2020). Moreover, smart products should move beyond functionality and incorporate emotional care elements—such as music, companionship features, or voice assistants that detect and respond to users’ emotional states—to promote psychological well-being (Curumsing et al., 2019). Emotional engagement through design can effectively enhance motivation and long-term adoption among older users.

Furthermore, elderly care service providers should strengthen their attention to emotional and social support. Beyond meeting daily living needs, community-based organizations can regularly organize cultural and recreational activities to help older adults expand their social networks and reduce feelings of isolation. At the same time, improving digital literacy through structured programs can empower older people to use smart products with greater confidence, creating a more inclusive digital environment that supports emotional well-being (Ngiam et al., 2022). Initiatives such as psychological counseling or peer support groups can further address emotional loneliness and foster a sense of belonging.

Finally, government efforts are essential to address the digital divide and support the emotional needs of older populations (State Council Office of the People’s Republic of China, 2020). Authorities should establish design standards for age-appropriate technologies, promote the development of simplified interfaces and functions, and encourage enterprises to consider the specific needs of different subgroups. In addition, maintaining offline service channels, simplifying digital service procedures, and offering financial subsidies or incentives can significantly improve access to smart technologies for older adults with limited digital skills or income. These policy-level interventions will help create a supportive digital environment where smart products and services can more effectively meet the emotional and practical needs of the aging population.

### 4.3 Research limitations and future research

Despite the study offering meaningful insights, several limitations should be acknowledged. The research was based on qualitative interviews with 20 relatively healthy and independent older adults living alone in urban Chengdu, recruited through non-random sampling. This limits the generalizability of the findings, as it excludes other subgroups such as rural residents, those with frail health, or those living with family members. The reliance on self-reported data and thematic analysis may also introduce subjectivity. Furthermore, due to cultural stigma and internalized shame, older adults may avoid expressing emotional vulnerability directly, often using neutral terms like “boredom” to describe deeper emotional distress (Grenade and Boldy, 2008), which suggests the need for carefully designed, culturally sensitive interview protocols. To enhance external validity and reliability,

## 5 Conclusion

This study contributes to a deeper understanding of the emotional needs of older people living alone (OPLA) in urban China and their complex relationship with smart technology. Through qualitative interviews, it reveals that emotional well-being among OPLA is closely tied to living arrangements and is often challenged by feelings of anxiety, loneliness, and diminished autonomy. While many older adults develop self-regulatory strategies such as engaging in leisure activities or maintaining social connections to cope with these challenges, their engagement with smart products remains limited due to low motivation, usability barriers, and the lack of age-sensitive design. These findings underscore the urgent need to shift from a purely functional view of aging technology to one that centers emotional responsiveness and cultural relevance. Future research should explore participatory and context-sensitive design strategies that embed emotional needs into product development. Furthermore, a collaborative effort among policymakers, designers, community organizations, and technology developers is essential to foster inclusive digital ecosystems that support emotional well-being, promote independent living, and enable active aging in a rapidly evolving technological landscape.

## Data Availability

The original contributions presented in the study are included in the article/supplementary material, further inquiries can be directed to the corresponding author/s.

## References

[ref1] Abell J. G. Steptoe A. (2021). Why is living alone in older age related to increased mortality risk? A longitudinal cohort study. Age Ageing 50, 2019–2024. doi: 10.1093/ageing/afab155, PMID: 34304269 PMC8675439

[ref9013] Astell A. J. McGrath C. Dove E. (2020). ‘That’s for old so and so’s!’: Does identity influence older adults’ technology adoption decisions? Ageing & Society 40, 1550–1576. doi: 10.1017/S0144686X19000230

[ref9010] Bandura A. (1997). Self-efficacy: The exercise of control. New York: W.H. Freeman.

[ref2] Banister J. Bloom D. E. Rosenberg L. (2012). “Population aging and economic growth in China” in The Chinese economy: A new transition. eds. Aoki M. Wu J. (London: Palgrave Macmillan UK).

[ref3] Borelli E. Paolini G. Antoniazzi F. Barbiroli M. Benassi F. Chesani F.. (2019). HABITAT: an IoT solution for independent elderly. Sensors 19:1258. doi: 10.3390/s19051258, PMID: 30871107 PMC6427271

[ref4] Braun V. Clarke V. (2006). Using thematic analysis in psychology. Qual. Res. Psychol. 3, 77–101. doi: 10.1191/1478088706qp063oa

[ref9008] Braun V. Clarke V. (2012). “Thematic analysis” in APA handbook of research methods in psychology. ed. Cooper H., vol. 2. Research designs (Washington, DC: American Psychological Association), 57–71.

[ref9003] British Geriatrics Society (BGS). (2017). Fit for frailty: Consensus best practice guidance. Available online at: https://www.bgs.org.uk/resources/resource-series/fit-for-frailty

[ref5] Buffel T. Phillipson C. Rémillard-Boilard S. (2019). “Age-friendly cities and communities: new directions for research and policy” in Encyclopedia of gerontology and population aging. eds. Danan G. Dupre M. E. (Cham: Springer International Publishing), 1–10.

[ref6] Carstensen L. L. Isaacowitz D. M. Charles S. T. (1999). Taking time seriously: a theory of socioemotional selectivity. Am. Psychol. 54, 165–181. doi: 10.1037/0003-066X.54.3.165, PMID: 10199217

[ref9011] Carstensen L. L. Pasupathi M. Mayr U. Nesselroade J. R. (2000). Emotional experience in everyday life across the adult life span. Journal of Personality and Social Psychology 79, 644–655. doi: 10.1037/0022-3514.79.4.644 11045744

[ref9012] Carstensen L. L. (2021). Socioemotional selectivity theory: The role of perceived endings in human motivation. The Gerontologist 61, 1188–1196. doi: 10.1093/geront/gnab116 34718558 PMC8599276

[ref7] Cattan M. White M. Bond J. Learmouth A. (2005). Preventing social isolation and loneliness among older people: a systematic review of health promotion interventions. Ageing Soc. 25, 41–67. doi: 10.1017/S0144686X04002594 27736564

[ref8] Charness N. Boot W. R. (2009). Aging and information technology use: potential and barriers. Curr. Dir. Psychol. Sci. 18, 253–258. doi: 10.1111/j.1467-8721.2009.01647.x

[ref9] Charpentier M. Kirouac L. (2022). Experiences of loneliness among older people living alone. A qualitative study in Quebec (Canada). Ageing Soc. 42, 2832–2853. doi: 10.1017/S0144686X21000349

[ref10] Chen X. Giles J. Yao Y. Yip W. Meng Q. Berkman L.. (2022). The path to healthy ageing in China: a Peking University-lancet commission. Lancet 400, 1967–2006. doi: 10.1016/S0140-6736(22)01546-X, PMID: 36423650 PMC9801271

[ref9001] Chengdu Municipal Bureau of Statistics (2020). Chengdu statistical yearbook 2020. Chengdu: Chengdu Municipal Bureau of Statistics.

[ref11] Chou K.-L. Chi I. (2000). Comparison between elderly Chinese living alone and those living with others. J. Gerontol. Soc. Work. 33, 51–66. doi: 10.1300/J083v33n04_05

[ref12] Cohen-Mansfield J. Perach R. (2015). Interventions for alleviating loneliness among older persons: a critical review. Am. J. Health Promot. 29, e109–e125. doi: 10.4278/ajhp.130418-LIT-182, PMID: 24575725

[ref13] Curumsing M. K. Fernando N. Abdelrazek M. Vasa R. Mouzakis K. Grundy J. (2019). Emotion-oriented requirements engineering: a case study in developing a smart home system for the elderly. J. Syst. Softw. 147, 215–229. doi: 10.1016/j.jss.2018.06.077

[ref9006] Denzin N. K. (1978). The research act: A theoretical introduction to sociological methods. 2nd Edn. New York: McGraw-Hill.

[ref14] Dermody G. Fritz R. Glass C. Dunham M. Whitehead L. (2021). Factors influencing community-dwelling older adults’ readiness to adopt smart home technology: a qualitative exploratory study. J. Adv. Nurs. 77, 4847–4861. doi: 10.1111/jan.14996, PMID: 34477222

[ref16] Fancourt D. Tymoszuk U. (2019). Cultural engagement and incident depression in older adults: evidence from the English longitudinal study of ageing. Br. J. Psychiatry 214, 225–229. doi: 10.1192/bjp.2018.267, PMID: 30560742 PMC6429253

[ref17] Fang E. F. Scheibye-Knudsen M. Jahn H. J. Li J. Ling L. Guo H.. (2015). A research agenda for aging in China in the 21st century. Ageing Res. Rev. 24, 197–205. doi: 10.1016/j.arr.2015.08.003, PMID: 26304837 PMC5179143

[ref9009] Friemel T. N. (2016). The digital divide has grown old: Determinants of a digital divide among seniors. New Media & Society 18, 313–331. doi: 10.1177/1461444814538648

[ref18] Ghorayeb A. Comber R. Gooberman-Hill R. (2021). Older adults’ perspectives of smart home technology: are we developing the technology that older people want? Int. J. Hum. Comput. Stud. 147:102571. doi: 10.1016/j.ijhcs.2020.102571, PMID: 40260412

[ref19] Grenade L. Boldy D. (2008). Social isolation and loneliness among older people: issues and future challenges in community and residential settings. Austral. Health Rev. 32:468. doi: 10.1071/AH080468 18666874

[ref20] Gu D. Feng Q. Yeung W.-J. J. (2019). Reciprocal dynamics of solo-living and health among older adults in contemporary China. J. Gerontol. 74, 1441–1452. doi: 10.1093/geronb/gby140, PMID: 30476326

[ref21] Guest G. Bunce A. Johnson L. (2006). How many interviews are enough: an experiment with data saturation and variability. Field Methods 18, 59–82. doi: 10.1177/1525822X05279903

[ref22] Han Y. He Y. Lyu J. Canqing Y. Bian M. Lee L. (2020). Aging in China: perspectives on public health. Global Health J. 4, 11–17. doi: 10.1016/j.glohj.2020.01.002

[ref23] Han A.-R. Park S.-A. Ahn B.-E. (2018). Reduced stress and improved physical functional ability in elderly with mental health problems following a horticultural therapy program. Complement. Ther. Med. 38, 19–23. doi: 10.1016/j.ctim.2018.03.011, PMID: 29857876

[ref25] Heinrich L. M. Gullone E. (2006). The clinical significance of loneliness: a literature review. Clin. Psychol. Rev. 26, 695–718. doi: 10.1016/j.cpr.2006.04.002, PMID: 16952717

[ref26] Hills P. Argyle M. (1998). Positive moods derived from leisure and their relationship to happiness and personality. Personal. Individ. Differ. 25, 523–535. doi: 10.1016/S0191-8869(98)00082-8

[ref27] Hong Y.-K. Wang Z.-Y. Cho J. Y. (2022). Global research trends on smart homes for older adults: bibliometric and Scientometric analyses. Int. J. Environ. Res. Public Health 19:14821. doi: 10.3390/ijerph192214821, PMID: 36429540 PMC9690352

[ref28] Hou B. Zhang H. (2023). Latent profile analysis of depression among older adults living alone in China. J. Affect. Disord. 325, 378–385. doi: 10.1016/j.jad.2022.12.154, PMID: 36640808

[ref29] Huang Y. Li X. Ling S. Zheng C. (2024). An analysis of how smart home product attributes influence older adults avoidance psychology: the sequential mediation role of product identity and trust. Behav. Sci. 14:1060. doi: 10.3390/bs14111060, PMID: 39594359 PMC11591175

[ref30] Jeste D. V. Lee E. E. (2019). Emerging empirical science of wisdom: definition, measurement, neurobiology, longevity, and interventions. Harv. Rev. Psychiatry 27, 127–140. doi: 10.1097/HRP.0000000000000205, PMID: 31082991 PMC6519134

[ref31] Jia Q. Duan Y. Gong R. Jiang M. You D. Yi Q. (2023). Living arrangements and depression of the older adults– evidence from the Chinese longitudinal healthy longevity survey. BMC Public Health 23:1870. doi: 10.1186/s12889-023-16730-4, PMID: 37759168 PMC10523833

[ref32] Kawamoto R. Yoshida O. Oka Y. Kodama A. (2005). Influence of living alone on emotional well-being in community-dwelling elderly persons. Geriatr Gerontol Int 5, 152–158. doi: 10.1111/j.1447-0594.2005.00285.x

[ref33] Kong D. Liu S. Hong Y. Chen K. Luo Y. (2023). Perspectives on the popularization of smart senior care to meet the demands of older adults living alone in communities of Southwest China: a qualitative study. Front. Public Health 11:1094745. doi: 10.3389/fpubh.2023.1094745, PMID: 36908438 PMC9998995

[ref34] Korstjens I. Moser A. (2017). Series: practical guidance to qualitative research. Part 2: context, research questions and designs. Eur. J. General Pract. 23, 274–279. doi: 10.1080/13814788.2017.1375090, PMID: 29185826 PMC8816399

[ref35] Kvale S. (1996). The 1,000-page question. Qual. Inq. 2, 275–284. doi: 10.1177/107780049600200302

[ref36] Lee G. R. Ishii-Kuntz M. (1987). Social interaction, loneliness, and emotional well-being among the elderly. Res. Aging 9, 459–482. doi: 10.1177/0164027587094001, PMID: 3438563

[ref37] Lee L. N. Kim M. J. (2020). A critical review of smart residential environments for older adults with a focus on pleasurable experience. Front. Psychol. 10. doi: 10.3389/fpsyg.2019.03080, PMID: 32038424 PMC6992566

[ref38] Li Y. (2013). A perspective on health Care for the Elderly who Lose Their Only Child in China. Scand. J. Public Health 41, 550–552. doi: 10.1177/1403494813490252, PMID: 23740862

[ref39] Li M. Woolrych R. (2021). Experiences of older people and social inclusion in relation to smart “age-friendly” cities: a case study of Chongqing, China. Front. Public Health 9:779913. doi: 10.3389/fpubh.2021.779913, PMID: 34988053 PMC8721664

[ref40] Lim L. L. Kua E.-H. (2011). Living alone, loneliness, and psychological well-being of older persons in Singapore. Current Gerontol. Geriatrics Res. 2011:e673181, 1–9. doi: 10.1155/2011/673181, PMID: 21969827 PMC3182578

[ref41] Lincoln Y. S. Guba E. G. (1985). Naturalistic inquiry, Beverly Hills, CA: Sage Publications. 438–439.

[ref42] Lou S. Liu H. (2023). Awareness, use, and need of smart Care for Older Adults: a comparative study based on a survey in Macao, China. Front. Public Health 11:1135164. doi: 10.3389/fpubh.2023.1135164, PMID: 37124815 PMC10141673

[ref43] Lucia-Palacios L. Pérez-López R. (2021). How can autonomy improve consumer experience when interacting with smart products? J. Res. Interact. Mark. 17, 19–37. doi: 10.1108/JRIM-02-2021-0031

[ref9005] Luo J. Lu C. Chen Y. Wu X. Zhu C. Cui W.. (2023). Nuclear translocation of cGAS orchestrates VEGF-A-mediated angiogenesis. Cell Reports 42:112328. doi: 10.1016/j.celrep.2023.112328 37027305

[ref44] Luo J.-D. Yeh Y.-C. (2012). Neither collectivism nor individualism: Trust in the Chinese Guanxi Circle. J. Trust Res. 2, 53–70. doi: 10.1080/21515581.2012.660355

[ref9004] Lou Y. Hong Y. (2023). Nash equilibrium computation in subnetwork zero-sum games with switching communications. IEEE Transactions on Automatic Control 68, 1234–1245. doi: 10.1109/TAC.2022.3145678

[ref45] Malatesta C. Z. Kalnok M. (1984). Emotional experience in younger and older adults. J. Gerontol. 39, 301–308. doi: 10.1093/geronj/39.3.301, PMID: 6715807

[ref46] Malone J. Dadswell A. (2018). The role of religion, spirituality and/or belief in positive ageing for older adults. Geriatrics 3:28. doi: 10.3390/geriatrics3020028, PMID: 31011066 PMC6319229

[ref47] Malterud K. Siersma V. D. Guassora A. D. (2015). Sample size in qualitative interview studies: guided by information power. Qual. Health Res. 26, 1753–1760. doi: 10.1177/1049732315617444 26613970

[ref48] Mellor D. Stokes M. Firth L. Hayashi Y. Cummins R. (2008). Need for belonging, relationship satisfaction, loneliness, and life satisfaction. Personal. Individ. Differ. 45, 213–218. doi: 10.1016/j.paid.2008.03.020

[ref49] Minichiello V. Aroni R. Hays T. N. (2008). In-depth interviewing: principles, techniques, analysis. Pearson Educ. Austral.

[ref9002] Mo J. (2010). The research on the elderly’s emotional needs in urban communities. Beijing: Social Sciences Academic Press.

[ref50] Morville P. (2005). Ambient findability: What we find changes who we become. Sebastopol, CA: O’Reilly Media, Inc.

[ref9014] Ngiam N. H. W. Yee W. Q. Teo N. Yow K. S. Soundararajan A. Lim J. X.. (2022). Building digital literacy in older adults of low socioeconomic status in Singapore (Project Wire Up): Nonrandomized controlled trial. J. Med. Inter. Res. 24:e40341. doi: 10.2196/40341 PMC9758632 36459398

[ref52] Norman D. A. (2004). Emotional design: Why we love (or hate) everyday things. New York, NY: Basic Books.

[ref53] Paredes M. Alejandra E. E. Lee L. C. Gupta S. Palmer B. W. Palinkas L. A.. (2021). Qualitative study of loneliness in a senior housing community: the importance of wisdom and other coping strategies. Aging Ment. Health 25, 559–566. doi: 10.1080/13607863.2019.1699022, PMID: 31918561 PMC7347442

[ref54] Patton M. Q. (1990). *Qualitative evaluation and research methods, 2nd ed.* qualitative evaluation and research methods. 2nd Edn. Thousand Oaks, CA, US: Sage Publications, Inc.

[ref55] Peek S. T. M. Wouters E. J. M. van Hoof J. Luijkx K. G. Boeije H. R. Vrijhoef H. J. M. (2014). Factors influencing acceptance of technology for aging in place: a systematic review. Int. J. Med. Inform. 83, 235–248. doi: 10.1016/j.ijmedinf.2014.01.004, PMID: 24529817

[ref56] Peine A. Marshall B. Martin W. Neven L. (2021). Socio-Gerontechnology: Interdisciplinary critical studies of ageing and technology. London: Routledge.

[ref57] Pérez-Escolar M. Canet F. (2023). Research on vulnerable people and digital inclusion: toward a consolidated taxonomical framework. Univ. Access Inf. Soc. 22, 1059–1072. doi: 10.1007/s10209-022-00867-x, PMID: 35125988 PMC8808275

[ref58] Raihan M. M. H. Subroto S. Chowdhury N. Koch K. Ruttan E. Turin T. C. (2024). Dimensions and barriers for digital (in)equity and digital divide: a systematic integrative review. Digital Transform. and Soc. 4, 111–127. doi: 10.1108/DTS-04-2024-0054

[ref59] Sandelowski M. (1995). Sample size in qualitative research. Res. Nurs. Health 18, 179–183. doi: 10.1002/nur.4770180211 7899572

[ref60] Sarid O. Melzer I. Kurz I. Shahar D. R. Ruch W. (2010). The effect of helping behavior and physical activity on mood states and depressive symptoms of elderly people. Clin. Gerontol. 33, 270–282. doi: 10.1080/07317115.2010.502105

[ref61] Saunders B. Sim J. Kingstone T. Baker S. Waterfield J. Bartlam B.. (2018). Saturation in qualitative research: exploring its conceptualization and operationalization. Qual. Quant. 52, 1893–1907. doi: 10.1007/s11135-017-0574-8, PMID: 29937585 PMC5993836

[ref63] Smith E. E. (2017). The power of meaning: The true route to happiness. London: River.

[ref64] Stanley M. Moyle W. Ballantyne A. Jaworski K. Corlis M. Oxlade D.. (2010). ‘Nowadays You Don’t even see your Neighbours’: loneliness in the everyday lives of older Australians. Health Soc. Care Community 18, 407–414. doi: 10.1111/j.1365-2524.2010.00923.x, PMID: 20491966

[ref65] State Council Office of the People’s Republic of China. (2020). Circular of the general Office of the State Council on the implementation plan for effectively addressing the difficulties of the elderly in the use of intelligent technology. Available online at: https://www.gov.cn/zhengce/content/2020-11/24/content_5563804.htm (Accessed July. 2024).

[ref67] Tohit N. Browning C. J. Radermacher H. (2012). ‘We want a peaceful life Here and hereafter’: healthy ageing perspectives of older Malays in Malaysia. Ageing Soc. 32, 405–424. doi: 10.1017/S0144686X11000316

[ref68] Tsai H.-y. S. Shillair R. Cotton S. R. Winstead V. Yost E. (2015). Getting grandma online: are tablets the answer for increasing digital inclusion for older adults in the U.S.? Educ. Gerontol. 41, 695–709. doi: 10.1080/03601277.2015.1048165, PMID: 26877583 PMC4748942

[ref69] Tsai H.-H. Tsai Y.-F. (2011). Changes in depressive symptoms, social support, and loneliness over 1 year after a minimum 3-month videoconference program for older nursing home residents. J. Med. Internet Res. 13:e1678:e93. doi: 10.2196/jmir.1678, PMID: 22086660 PMC3222194

[ref51] United Nations. (2020). The sustainable development goals report 2020. New York: United Nations. Available online at: https://unstats.un.org/sdgs/report/2020/ (Accessed January 31, 2024)

[ref70] Vaismoradi M. Turunen H. Bondas T. (2013). Content analysis and thematic analysis: implications for conducting a qualitative descriptive study. Nurs. Health Sci. 15, 398–405. doi: 10.1111/nhs.12048, PMID: 23480423

[ref71] Vaportzis E. Clausen M. G. Gow A. J. (2017). Older adults perceptions of technology and barriers to interacting with tablet computers: a focus group study. Front. Psychol. 8:01687. doi: 10.3389/fpsyg.2017.01687, PMID: 29071004 PMC5649151

[ref72] Venkatesh V. Morris M. G. Davis G. B. Davis F. D. (2003). User acceptance of information technology: toward a unified view. MIS Q. 27, 425–478. doi: 10.2307/30036540

[ref9007] Victor C. R. Yang K. (2012). The prevalence of loneliness among adults: A case study of the United Kingdom. The. Journal of Psychology: Interdisciplinary and Applied 146, 85–104. doi: 10.1080/00223980.2011.613875 22303614

[ref73] Wang Y. (2012). Urban development and urban planning in contemporary China: A case study of Chengdu. Montana State University - Bozeman, College of Letters & Science. Available online at: https://scholarworks.montana.edu/xmlui/handle/1/2500 (Accessed July 30, 2024).

[ref74] Wang J. Chen T. Han B. (2014). Does co-residence with adult children associate with better psychological well-being among the oldest old in China? Aging Ment. Health 18, 232–239. doi: 10.1080/13607863.2013.837143, PMID: 24053437 PMC4158609

[ref76] Wang C.-M. Tseng S.-M. Huang C.-S. (2020). Design of an Interactive Nostalgic Amusement Device with user-friendly tangible interfaces for improving the health of older adults. Healthcare 8:179. doi: 10.3390/healthcare8020179, PMID: 32575389 PMC7349908

[ref77] Wang J. Yang Q. Bei W. (2023). Effects of care arrangement on the age of institutionalization among community-dwelling Chinese older adults. J. Aging Soc. Policy 35, 595–610. doi: 10.1080/08959420.2020.1726720, PMID: 32033523

[ref78] Wang Y. Zeng H. Lv F. Wang J. (2024). Analysis of demand and influencing factors for smart senior care among older adults in underdeveloped regions of Western China: a case study of Lanzhou. Front. Public Health 12:1337584. doi: 10.3389/fpubh.2024.1337584, PMID: 38939563 PMC11210194

[ref79] Weinert C. Cudney S. Hill W. G. (2008). Rural women, technology, and self-Management of Chronic Illness. Canad. J. Nurs. Res. 40, 114–134, PMID: 18947095 PMC2700733

[ref80] Whear R. Campbell F. Rogers M. Sutton A. Robinson-Carter E. Sharpe R.. (2023). What is the effect of intergenerational activities on the wellbeing and mental health of older people?: a systematic review. Campbell Syst. Rev. 19:e1355. doi: 10.1002/cl2.1355, PMID: 37795424 PMC10546258

[ref81] You K. S. Lee H. O. (2006). The physical, mental, and emotional health of older people who are living alone or with relatives. Arch. Psychiatr. Nurs. 20, 193–201. doi: 10.1016/j.apnu.2005.12.008, PMID: 16846780

[ref82] Zhang S. Li X. Yang M. Song H. (2023). Older adults’ heterogeneous preferences for climate-proof urban blue-green spaces: a case of Chengdu, China. Urban For. Urban Green. 90:128139. doi: 10.1016/j.ufug.2023.128139

